# Vascular Endothelial Glycocalyx Damage in COVID-19

**DOI:** 10.3390/ijms21249712

**Published:** 2020-12-19

**Authors:** Minako Yamaoka-Tojo

**Affiliations:** 1 Department of Rehabilitation/Regenerative Medicine and Cell Design Research Facility, Kitasato University School of Allied Health Sciences, Sagamihara 252-0373, Japan; myamaoka@med.kitasato-u.ac.jp; Tel.: +81-42-778-8111; Fax: +81-42-778-9696; 2 Department of Cardiovascular Medicine, Kitasato University Graduate School of Medical Sciences, Sagamihara 252-0373, Japan

**Keywords:** vascular endothelial dysfunction, COVID-19, systemic inflammatory-reactive microvascular endotheliopathy (SIRME), syndecan-1, vascular endothelial glycocalyx (VEGLX)

## Abstract

The new coronavirus disease-2019 (COVID-19), which is spreading around the world and threatening people, is easily infecting a large number of people through airborne droplets; moreover, patients with hypertension, diabetes, obesity, and cardiovascular disease are more likely to experience severe conditions. Vascular endothelial dysfunction has been suggested as a common feature of high-risk patients prone to severe COVID-19, and measurement of vascular endothelial function may be recommended for predicting severe conditions in high-risk patients with COVID-19. However, fragmented vascular endothelial glycocalyx (VEGLX) is elevated in COVID-19 patients, suggesting that it may be useful as a prognostic indicator. Although the relationship between VEGLX and severe acute respiratory syndrome coronavirus 2 infections has not been well studied, some investigations into COVID-19 have clarified the relationship between VEGLX and the mechanism that leads to severe conditions. Clarifying the usefulness of VEGLX assessment as a predictive indicator of the development of severe complications is important as a strategy for confronting pandemics caused by new viruses with a high affinity for the vascular endothelium that may recur in the future.

## 1. Introduction

The new coronavirus disease-2019 (COVID-19) has disrupted society, accelerated economic damage, and poses a threat to the population due to insufficient information on its mode of transmission and pathogenesis. A year after the start of the COVID-19 pandemic outbreak, the identity of the causative virus, severe acute respiratory syndrome coronavirus 2 (SARS-CoV-2), has become more or less clear, as various studies on the disease have been reported from around the world. Findings of particular importance are: (1) the virus is highly contagious and can easily spread to a large number of people via droplets from their mouth; (2) it can be prevented by maintaining social distancing and avoiding confined spaces; and (3) although most infected people would only have a mild illness, it is not uncommon in the elderly, and in smokers. Moreover, patients with hypertension, diabetes, obesity, and cardiovascular diseases are more likely to be severely affected by COVID-19 and have a higher mortality rate. These three findings provide strategies for combating COVID-19.

Vascular endothelial damage has been identified as a common feature of high-risk patients prone to severe COVID-19 [1]. According to a report about 7 lungs obtained during autopsy from patients who died from SARS-CoV-2 infection, severe endothelial injury was found to be associated with intracellular SARS-CoV-2 virus with disrupted endothelial cell membrane [2]. The lungs from the patients with COVID-19 exhibited widespread vascular thrombosis with microangiopathy and occlusion of alveolar capillaries, and significant new vessel growth through a mechanism of intussusceptive angiogenesis [2]. On the other hand, the vascular endothelial glycocalyx (VEGLX), the extracellular matrix covering vascular endothelial cells throughout the body, is also impaired by various risk factors, and there have been numerous reports of VEGLX damage in acute respiratory distress syndrome (ARDS) and disseminated intravascular coagulation (DIC), which may be closely related to vascular endothelial damage in severe COVID-19. Recently, circulating levels of fragmented VEGLX concentrations and real-time measurements of VEGLX in sublingual capillaries have been reported in patients with COVID-19 [3].

This review discusses existing reports on COVID-19 and VEGLX damage along with their mechanisms.

## 2. Coronavirus Infections

Seven coronavirus (CoV) infections have been identified to infect humans so far, with 4 conventional cold viruses and 3 severe ones. Among them, 15–20% of common colds are caused by conventional human coronavirus (HCoV) infections induced by HCoV-229E, HCoV-OC43, HCoV-HKU1, and HCoVNL63. In the winter epidemic season, approximately 30% of common colds are caused by conventional HCoV. The viruses that cause pandemics include SARS-CoV, Middle East respiratory syndrome (MERS)-CoV, and SARS-CoV-2, the virus causing COVID-19.

It has been suggested that influenza infections and SARS/MERS/COVID-19 are predisposed to cause a pandemic because these infections are zoonotic and can be transmitted to and from non-human animals. This is because the virus can multiply and mutate in various animals, potentially mutating into a virus that is more infectious and more likely to cause severe disease in humans. Since these viruses are expected to continue to mutate, we anticipate that we will need to be vaccinated with a multivalent vaccine every season to prevent a pandemic.

**Figure 1:**
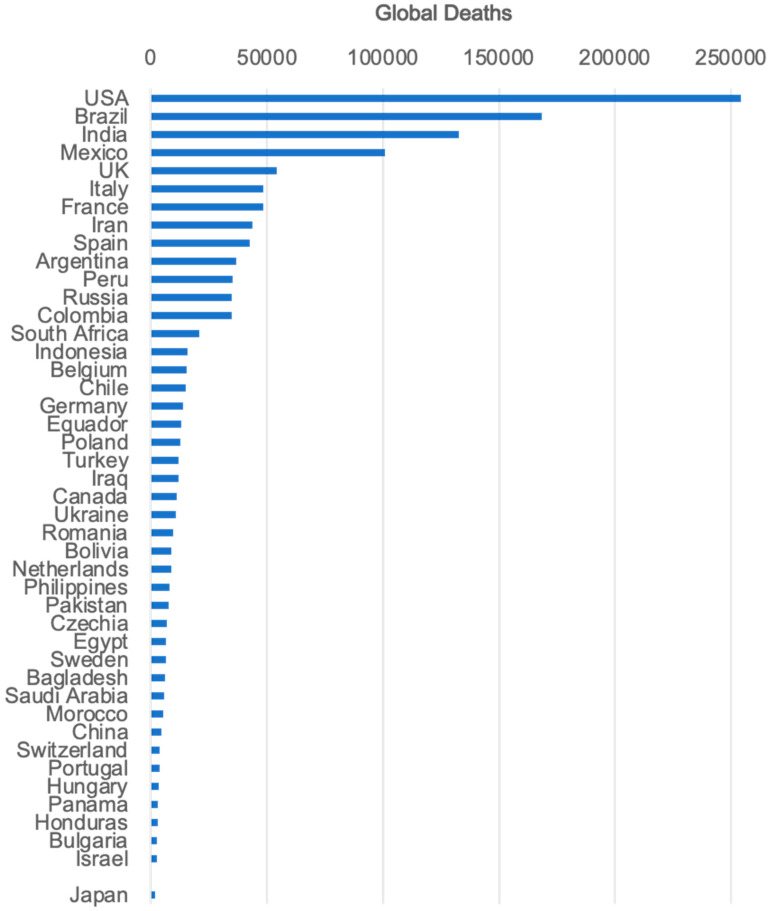
The number of global deaths due to the new coronavirus disease-2019 (COVID-19). The total number of global deaths due to COVID-19 was 1,372,182 as of 21 November 2020. The graph was generated from the COVID-19 Dashboard website by the Center for Systemics Science and Engineering at Johns Hopkins University (https://systems.jhu.edu. Last updated on 21 November 2020).

Compared to SARS/MERS, COVID-19 has a lower mortality rate and a higher proportion of mild cases, resulting in a situation in which the virus is spreading worldwide (Figure 1). According to the COVID-19 Dashboard website by the Center for Systemics Science and Engineering at the Johns Hopkins University, Baltimore, MD, the number of global deaths due to COVID-19 was 1,527,209 as of 6 December 2020.

In a recent multicenter report of 3894 COVID-19 patients from Italy, impaired renal function, elevated C-reactive protein, and advanced age were major predictors of in-hospital death [4]. Some of these risk factors of severe COVID-19 may vary according to sex, age, and geographical location for reasons that are not fully understood. These factors are possibly associated with the timing of the pandemic outbreak and the local management modalities of patients.

The current treatments for COVID-19 recommended in Japan are remdesivir and dexamethasone, or extracorporeal membrane oxygenation (ECMO) in most severely injured patients. Other drugs such as tocilizumab and favipiravir are also expected to be effective and are undergoing clinical research [5]. Although the long-term effects of COVID-19 are not yet known, the unfavorable effects of systemic microvascular damage induced by COVID-19 may promote fibrosis of various organs later. Future progression of atherosclerosis, heart failure with impaired diastolic function, Kawasaki disease-like coronary aneurysms, and systemic vasculitis may be possible sequelae.

## 3. Spike Domain in Coronavirus

**Figure 2:**
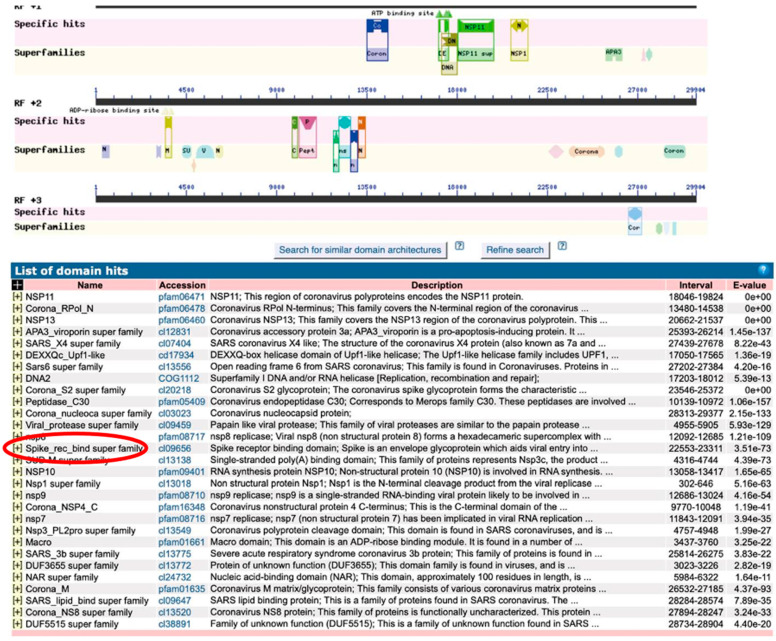
**Severe acute respiratory syndrome coronavirus 2** (SARS-CoV-2) domain search. Results were obtained from the Basic Local Alignment Search Tool (BLAST) domain search of amino acid sequences translated from the genome sequence of the virus responsible for COVID-19 on 15 February 2020. The “Spike_rec_bind superfamily” domain, which is the 14th from the top, is called the spike receptor-binding super family and is described as binding to ACE2 angiotensin-converting enzyme 2 (ACE2). At that time, it was listed as “Wuhan seafood market pneumonia virus isolate Wuhan-Hu-1, complete genome” (https://www.ncbi.nlm.nih.gov/nuccore/NC_045512). As of 22 November 2020, it is listed as “Severe acute respiratory syndrome coronavirus 2 isolate Wuhan-Hu-1, complete genome”.

Figure 2 shows the results of the Basic Local Alignment Search Tool (BLAST) domain search that yielded amino acid sequences translated from the genome sequence of the virus (later named SARS-CoV-2) responsible for COVID-19 on 15 February 2020. At that time, it was described as “Wuhan seafood market pneumonia virus isolate Wuhan-Hu-1, complete genome” (https://www.ncbi.nlm.nih.gov/nuccore/NC_045512). As of 22 November 2020, it is listed as “Severe acute respiratory syndrome coronavirus 2 isolate Wuhan-Hu-1, complete genome” The “Spike_rec_bind super family” domain, which is 14th from the top, is called the spike receptor-binding super family and is involved in binding to angiotensin-converting enzyme 2 (ACE2).

A common structure among coronaviruses is the spike, which is reminiscent of the corona from which it takes its name. This structure is characteristic of the coronavirus group, which has a spike receptor-binding domain. This spike is composed of an envelope (outer membrane) glycoprotein that assists the entry of the virus into the host cell. This domain binds to ACE2 [6,7], which allows membrane adhesion between the host cell membrane and the envelope and controls the delivery of viral genes and proteins within the envelope into the host cell.

As a consequence of increased gene expression of ACE2 and the proteolytic enzyme transmembrane protease serine 4 (TMPRSS4) in bronchial epithelial cells of smokers [8,9], smokers may be more easily and severely infected with SARS-CoV-2. In addition, smokers, the elderly, and patients with hypertension, heart failure, and atherosclerotic disease have been found to exhibit increased expression of ACE2 genes and proteins in vascular endothelial cells [10]. The increased expression of ACE2 in these subjects may be due to a biological defense mechanism to protect blood vessels from damage [11]. In such patients, there is a concurrent VEGLX damage. Vascular endothelial cells are more susceptible to infection with SARS-CoV-2 and are likely hosts for viral proliferation, and as a consequence may be more prone to progression of severe COVID-19. On the other hand, the ACE2 gene is under-expressed in the nasal mucosa of children younger than 10 years of age [12], which may be one reason why SARS-CoV-2 infection is relatively unlikely to occur in children [13].

**Figure 3:**
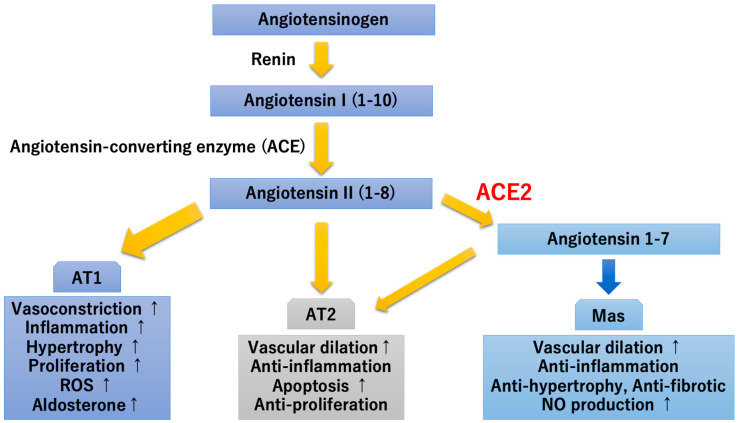
Renin-angiotensin system and angiotensin-converting enzyme 2 (ACE2). ACE2 was identified as an enzyme that degrades angiotensin II to angiotensin 1–7, which binds to the Mas receptor. The ACE2/angiotensin 1–7/Mas axis is an important repressor of the renin-angiotensin system. (↑) shows upregulation. AT1, angiotensin type 1 receptor; AT2, angiotensin type 2 receptor; Mas, G protein-coupled proto-oncogene Mas receptor; ROS, reactive oxygen species; NO, nitric oxide.

SARS-CoV-2 has a strong affinity for ACE2-rich vascular endothelial cells, which may be one of the reasons why severe complications associated with endothelial damage are more likely, especially in patients with hypertension, diabetes, cardiovascular disease, smokers, and obese individuals expressing high levels of ACE2. Membrane-bound aminopeptidase ACE2 plays an important role in inflammation, which could be attributed to the conversion of the pro-inflammatory angiotensin II peptide into angiotensin 1–7, a peptide that opposes the actions of angiotensin II (Figure 3). Mas, which is thought to be a proto-oncogene, was reported to act as a receptor for angiotensin 1–7. Since then, the ACE2/angiotensin 1–7/Mas receptor axis has attracted attention as a repressor of the renin-angiotensin system in heart failure, hypertension, diabetes, and atherosclerosis [14]. It has been reported that the induction of diabetes in ACE2-deficient mice accelerates the worsening of nephropathy and that ACE2 deficiency exacerbates insulin sensitivity in mice and the development of atherosclerosis and cardiac remodeling [15,16].

The ACE2 was also identified as a functional receptor for coronaviruses, including SARS-CoV and SARS-CoV-2 [17]. When surface-expressed spike proteins (S proteins) of SARS-CoV-2 bind to the transmembrane protein ACE2 on the cell membrane, they are cleaved by TMPRSS2, which allows the S proteins to be activated [18]. Activation of the S protein is important for the entry of the virus into cells by fusion of the viral outer membrane with the cell membrane. Furthermore, ACE2 deficiency, which could be induced by binding with SARS-CoV-2, is considered to drive a cytokine storm in patients with COVID-19 [19]. 

## 4. Systemic Inflammatory-Reactive Microvascular Endotheliopathy (SIRME)

**Figure 4:**
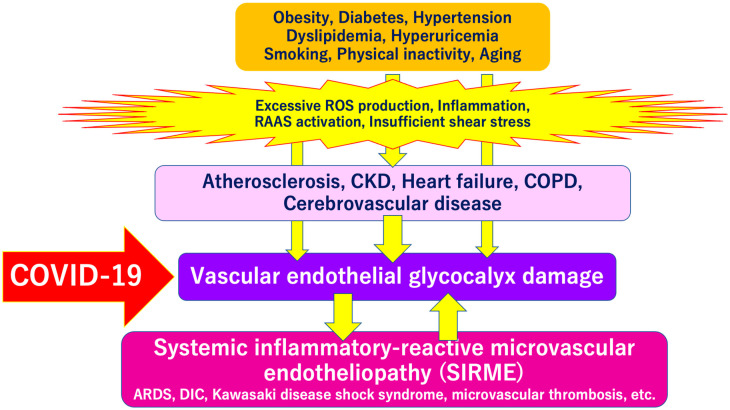
Severe COVID-19 comorbidity induced by vascular endothelial glycocalyx (VEGLX) damage. The VEGLX is damaged due to various factors such as smoking, physical inactivity, hypertension, diabetes, obesity, and cardiovascular diseases. Various lethal conditions in COVID-19 (e.g., acute respiratory distress syndrome (ARDS), disseminated intravascular coagulation (DIC), Kawasaki disease shock syndrome, microvascular thrombosis, etc.) may be caused by a common mechanism, damage of VEGLX. ROS, reactive oxygen species; RAAS, renin-angiotensin-aldosterone system; CKD, chronic kidney disease; COPD, chronic obstructive pulmonary disease.

SARS-CoV-2 directly invades vascular endothelial cells and causes systemic inflammatory microvascular endothelial disorders such as leakage of plasma components from microvessels, intramicrovascular blood clotting and thrombus formation, and excessive release of inflammatory cytokines following vascular endothelial dysfunction [16]. It might play a central role in the pathogenesis of ARDS [20] and multi-organ failure [21,22,23]. Such a wide variety of serious pathologies can be explained by the concept of “systemic inflammation-reactive microvascular endotheliosis (SIRME)”, in which the VEGLX is rapidly and systemically disrupted (Figure 4).

On the other hand, extensive VEGLX damage has been reported to occur in patients with cardiovascular disease and its risk factors, suggesting that SARS-CoV-2 can easily penetrate endothelial cells in impaired vascular endothelial cells with a loss of barrier function, causing severe COVID-19 [24].

## 5. Vascular Endothelial Glycocalyx (VEGLX)

**Figure 5:**
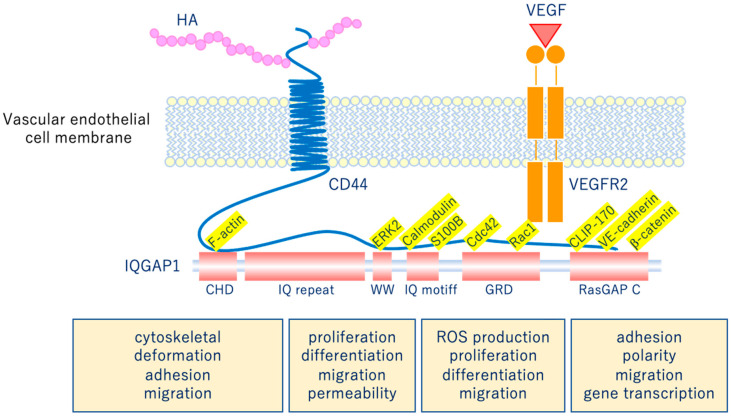
Vascular endothelial glycocalyx regulates cellular functions. Hyaluronic acid (HA) activates the HA/CD44 system by binding to CD44 and regulates various intracellular signaling through the multifunctional platform IQGAP1, as well as the vascular endothelial growth factor VEGF/VEGFR2 system. VEGF, vascular endothelial growth factor; VEGFR2, VEGF receptor 2; CD44, cluster of differentiation 44; CHD, calponin homology domain; IQ repeat, IQGAP specific repeat; ERK, extracellular-signal-regulated kinase; WW, region containing two tryptophans; S100B, S100 calcium-binding protein B; IQ motif, calmodulin-binding motif; Cdc42, cell division cycle 42; Rac1, Rac family small GTPase 1; GRD, Ras GTPase-activating protein-related domain; CLIP-170, cytoplasmic linker protein 170; RasGAP C, Ras GTPase-activating protein C terminus; ROS, reactive oxygen species.

The glycocalyx is a complex gel-like layer of glycosylated lipid-protein mixtures that covers the surface of all living cells, and is known to serve as a physical protective layer as well as a buffer region between cells and the extracellular matrix to control various cellular functions [25]. Vascular endothelial cells are a monolayer of cells that comprise the innermost layer of cells in the vascular system, including arteries, veins, and capillaries, and serve a barrier function for the blood vessels surrounding all organs and in direct contact with the blood flowing through the vascular lumen. As shown in Figure 5, for example, the VEGLX plays an important role in regulating various vascular endothelial cell functions such as coagulation, inflammation, vasoconstriction and relaxation, vascular permeability, and angiogenesis [26,27].

**Figure 6:**
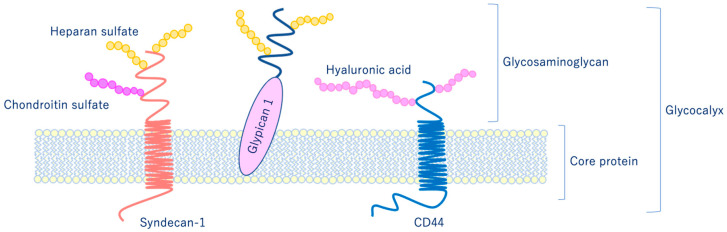
Schematic representation of the vascular endothelial glycocalyx, which is present in the vascular endothelial cell membrane. The vascular endothelial glycocalyx is composed of core proteins and highly water-retaining glycosaminoglycans that bind to the cell membrane.

As shown in Figure 6, glycocalyx consists of sialic acid-containing glycoproteins, core proteins consisting of membrane-bound proteoglycans (syndecan, glypican, etc.), glycosaminoglycan side chains (heparan sulfate, chondroitin sulfate, etc.), and long-chain hyaluronic acid (HA) [28,29]. VEGLX is stabilized by shear stress [30] and this stabilization is crucial for nitric oxide (NO) production in vascular endothelial cells [31,32]. Although glycosaminoglycans are constantly degraded by enzymes, their levels are maintained by newly synthesized glycosaminoglycans supplied from vesicles in the golgi apparatus, regulating their homeostatic balance [33].

**Figure 7:**
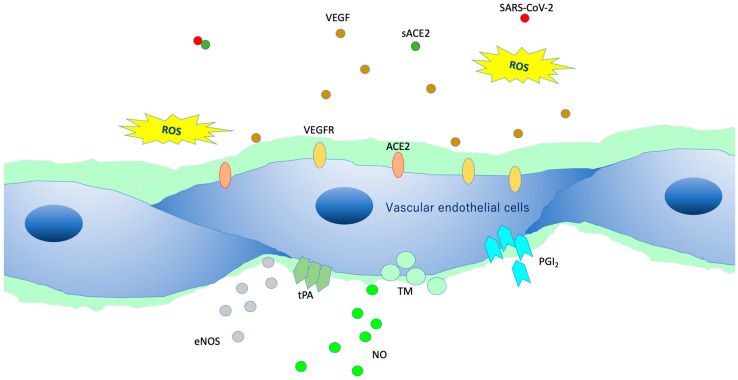
Intact vascular endothelial glycocalyx (VEGLX). When vascular endothelial cells are covered enough with healthy VEGLX, even if severe acute respiratory coronavirus 2 (SARS-CoV-2) enters the body, it could be neutralized by the effects of appropriate reactive oxygen species (ROS) and soluble angiotensin-converting enzyme 2 (sACE2), and it may be possible to prevent the virus entry into the vascular endothelium. VEGF, vascular endothelial growth factor; VEGFR, VEGF receptors; NO, nitric oxide; eNOS, endothelial NO synthase; TM, thrombomodulin; tPA, tissue plasminogen activator; PGI2, prostacyclin.

VEGLX protects vascular endothelial cells from the turbulence caused by blood flow and is involved in the regulatory function of the vascular permeability barrier. In addition, it plays an important role in endothelial function, especially in microvascular endothelial function, as it controls vascular reactivity and is involved in regulating the interaction between vascular endothelial cells and blood components [34]. Vascular endothelial cells in a healthy state covered by the VEGLX (Figure 7) contain a variety of cytokines, chemokines, receptors, growth factors, gap-binding proteins, extracellular superoxide dismutase (ecSOD), and endothelial nitric oxide synthase (eNOS). In addition, lipoprotein lipases are expressed that play a variety of functions required for homeostasis. Owing to its negative charge, VEGLX is also thought to be a determinant of salt sensitivity, and neutralizes cell surface charges by retaining plasma sodium in the glycocalyx layer [35].

## 6. Damage of VEGLX

**Figure 8:**
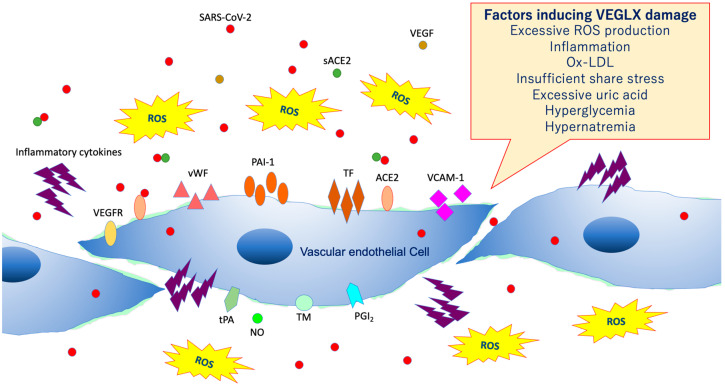
Damaged vascular endothelial glycocalyx (VEGLX). VEGLX damage is associated with vascular endothelial dysfunction, which induces reduced nitric oxide (NO) bioavailability, increased excessive reactive oxygen species (ROS) production, inflammatory cytokine release, platelet adherence, coagulation, and leukocyte adhesion. SARS-CoV-2, severe acute respiratory coronavirus 2; VEGF, vascular endothelial growth factor; VEGFR, VEGF receptor; ACE2, angiotensin-converting enzyme 2; sACE2, soluble ACE2; PAI-1, plasminogen activator inhibitor-1; TF, tissue factor; vWF, von Willebrand factor; ox-LDL, oxidized low-density lipoprotein; MMPs, matrix metalloproteases; tPA, tissue plasminogen activator; PGI2, prostacyclin; TM, thrombomodulin.

As shown in Figure 8, VEGLX shedding and degradation are known to be caused by a variety of cellular stresses [36]. Specifically, ischemia/reperfusion injury [37], endotoxin [38], inflammatory mediators [39], atrial natriuretic peptide, hypoxia, excessive reactive oxygen species (ROS), uric acid [40], hyperglycemia [41,42], hypernatremia [43], excessive fluid infusion, dehydration, decreased vascular wall shear stress, oxidized low-density lipoprotein (ox-LDL) [44], and others can cause VEGLX damage. In addition, this damage is known to be sex-specific, mostly observed in men. Systemic shedding of VEGLX has been associated with serious infections [45], Kawasaki disease [46], gestational hypertensive nephropathy (preeclampsia) [47], gestational diabetes [48], sepsis [49], acute lung injury (ALI)/ARDS [50], trauma [51], ischemic cerebral embolism [52], acute coronary syndrome [53], and shock [54]. The term shock-induced endothelial glycocalyx disturbance, called shock-induced endotheliopathy (SHINE), is an indicator of poor prognosis in fairly serious conditions, such as severe trauma, sepsis, myocardial infarction, and cardiac arrest syndrome [55]. Under these severe conditions, VEGLX is degraded via inflammatory mechanisms that promote tissue degradation, including reactive activation of metalloproteinases, heparanases, and hyaluronidases [23,56]. In patients with these acute diseases, high concentrations of fragmented VEGLX, such as syndecan-1 (soluble CD138), syndecan-4, hyaluronic acid, and heparan sulfate, can be detected in the blood. The degraded VEGLX detaches from the surface of vascular endothelial cells, thinning the glycocalyx layer, and inducing extravascular leakage in microvessels with excessive vascular permeability, contributing to further pathological deterioration by causing interstitial edema in various organs [56,57].

## 7. Evaluation of VEGLX In Vivo

**Figure 9:**
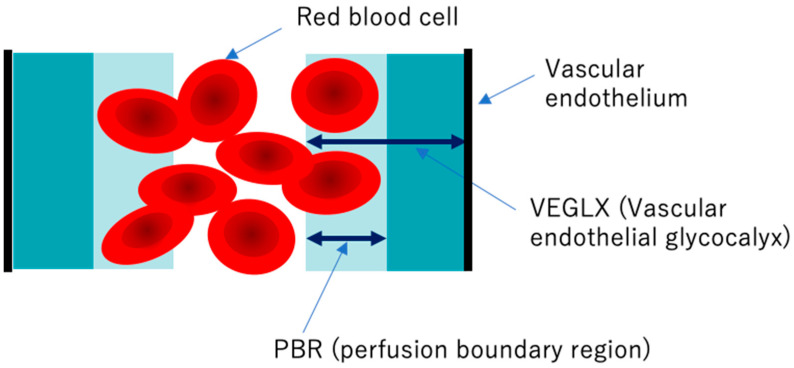
Conceptual diagram of the vascular endothelial glycocalyx (VEGLX) vulnerable region (PBR). The VEGLX, which covers the surface of vascular endothelial cells, can be broadly divided into a region of dense glycans on the vascular endothelial side and a region covered by fragile glycans on the vascular lumen side. In disorders of the VEGLX, the fragile region is known to be enlarged.

Elevated concentrations of soluble VEGLX fragments in the blood of patients with sepsis and diabetes have been previously reported and qualitatively assessed using electron micrographic imaging of the microvascular VEGLX in animal models and autopsy cases. The endothelial glycocalyx measurement device has emerged as a simple and reproducible instrument for measuring VEGLX by monitoring the sublingual microcirculation of the VEGLX vulnerable region as an indicator of the perfusion boundary region (PBR). Evaluation of the VEGLX using GlycoCheck^®^ (Microvascular Health Solutions, UT, USA) has been used [58]. The VEGLX is composed of a tight glycocalyx layer and a coarse glycocalyx layer. The latter allows red blood cells to bounce freely within the layer, which is observed as an increased PBR (Figure 9).

To detect VEGLX damage, PBR of the sublingual arterial microvessels is measured as a noninvasive test. Higher PBR values are thought to indicate an increase in this vulnerable area of the VEGLX and represent a thinning of the tight healthy VEGLX. In addition to reports on physiological changes in the VEGLX in healthy subjects, there have been reports on PBR related to a variety of diseases. A higher PBR was observed in elderly people, women, people with low body mass index (BMI), and patients with diastolic hypertension and diabetes [59,60,61]. In the context of racial differences, one report found no obvious racial differences between 472 Chinese and 254 Flemish residents [62], while a cross-sectional report of 6169 people found racial differences in allowing for increased PBR among blacks of African descent and whites of European descent compared to Asians and Arabs [59].

In diseases associated with complications of severe coronavirus, infections include sepsis, chronic heart failure [63], cerebrovascular disease [64], microvascular angina [65], and ischemic heart disease [64], PBR has been reported to be associated with the severity of these diseases, suggesting the usefulness of VEGLX measurements [66,67].

## 8. Virus Infections and VEGLX

SARS-CoV-2, the virus responsible for COVID-19, is a positive-sense single-stranded RNA virus that is classified as a (+)ssRNA virus. The genome of positive-stranded RNA viruses also acts as mRNA and is translated into proteins in the host cell. These viruses are categorized into Group IV of the Baltimore Classification of Viruses, and are classified as dengue fever virus, and include SARS-CoV-1, MERS-CoV2, hepatitis C virus, yellow fever virus, Japanese encephalitis viruses, and rhinoviruses.

In case of viral infections, research on dengue fever and VEGLX has been reported. Dengue hemorrhagic fever/dengue shock syndrome (DHF/DSS) is characterized by microvascular barrier dysfunction and shock [68]. Dengue virus nonstructural protein 1 (NS1) has been identified as the only membrane-associated protein that anchors the viral replication complex to the vascular endothelial cell membrane. Increased blood levels of VEGLX components such as hyaluronic acid, heparin sulfate, claudin-5, and syndecan-1, suggesting shedding of the VEGLX and extravascular leakage of plasma components, have been associated with the development and exacerbation of severe dengue infection [69,70]. Such viral entry into and proliferation of vascular endothelial cells in severe viral infections is responsible for a variety of microvascular disorders, including ARDS and DIC [71,72].

The susceptibility of influenza A virus to host cell infection is defined by the density, glycosylation state, and length of the glycocalyx, which constitutes a protective barrier called mucin on the surface of the cell [73]. Dense mucin is the most important factor in the ability of influenza virus to penetrate the cell surface and inhibit binding to glycolipid receptors. The rate at which the virus fuses to the cell is found slower in a concentration-dependent manner with respect to mucin [73]. Several flu drugs target the interaction between the influenza virus and mucin-like proteins [73].

## 9. Definition of SIRME

**Figure 10:**
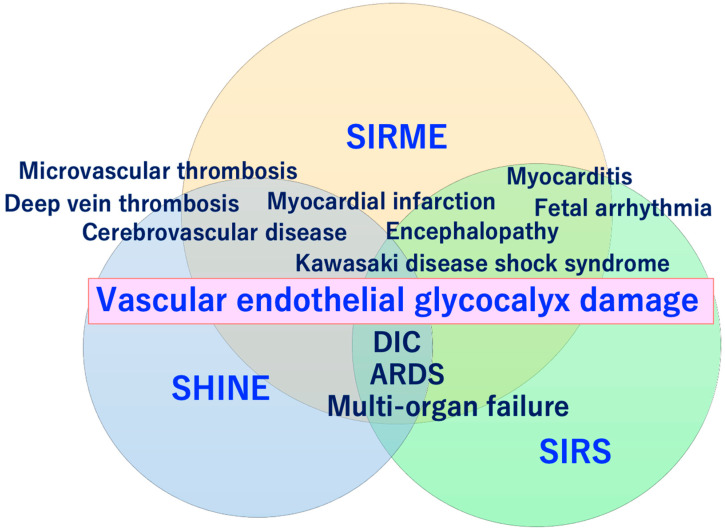
Conceptual diagram showing the relationship between shock-induced endotheliopathy (SHINE)/systemic inflammatory-reactive syndrome (SIRS) and the vascular endothelial glycocalyx (VEGLX) damage that causes systemic inflammation-reactive microvascular endotheliopathy (SIRME). SIRME is caused by a systemic disorder of the VEGLX, resulting in extravascular leakage of plasma components, increased thrombogenicity, increased production of reactive oxygen species, and an excess state of inflammatory cytokines, leading to microvascular embolism, venous thrombosis, and Kawasaki disease shock syndrome. As the disease worsens, it progresses to severe SIRME, including the concept of SIRS and SHINE, leading to severe conditions such as disseminated intravascular coagulation (DIC) and acute respiratory distress syndrome (ARDS).

VEGLX disorders associated with sympathetic and adrenal hyperfunction, similar to shock-induced endotheliosis (SHINE) observed in extremely severe conditions such as post-cardiac arrest syndrome, also occur in COVID-19, which does not meet the criteria for systemic inflammatory response syndrome (SIRS), and in non-shock conditions. It is also a condition observed in patients with mild to moderate COVID-19. For this reason, the newly proposed systemic inflammation-reactive microvascular endotheliosis (SIRME) caused by SIRS-CoV-2 as a mechanism for the development of various complications caused by COVID-19 is shown in Figure 10.

**Figure 11:**
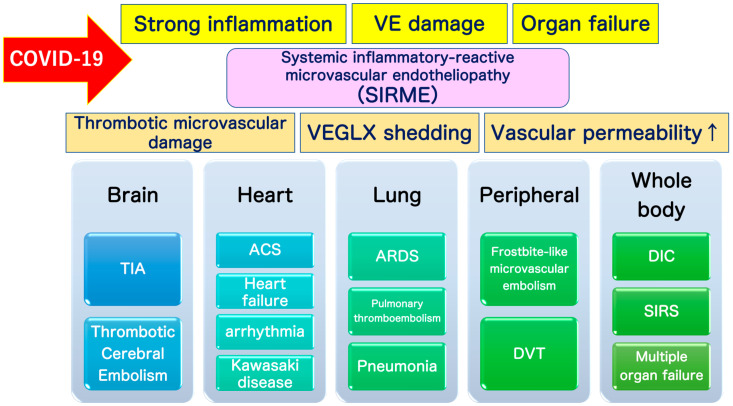
Definition of systemic inflammatory-reactive microvascular endotheliopathy (SIRME). SIRME is caused by damage to the vascular endothelial glycocalyx (VEGLX), which is impaired in an inflammatory response. SIRME is characterized by (1) the presence of causative strong inflammation, (2) vascular endothelial damage with strong thrombogenic tendency and increased vascular permeability, (3) organ failure. SIRME is presumed to be one of the major mechanisms causing diverse complications of COVID-19. (↑) shows upregulation. VE, vascular endothelial; VEGLX, vascular endothelial glycocalyx; TIA, transient ischemic attack; ACS, acute coronary syndrome; ARDS, acute respiratory distress syndrome; DVT, deep vein thrombosis; DIC: disseminated intravascular coagulation; SIRS, systemic inflammatory response syndrome.

The VEGLX disorder as its primary mechanism, characterized by (1) thrombotic microvascular disease, (2) shedding of the VEGLX, and (3) increased vascular permeability [24]. Pathologies caused by SIRME include (1) the presence of causative inflammation (fever, high levels of C-reactive protein and proinflammatory cytokines), (2) vascular endothelial damage with strong thrombogenic tendencies (high D-dimer and FDP) and increased vascular permeability; and (3) organ damage (increased respiratory rate, high levels of lactate dehydrogenase and transaminases, and elevated myocardial deviation enzymes), and are defined as a condition in which all 3 pathologies are present at the same time (Table 1). In addition to (1) to (3), the presence of (4) abnormally high blood levels of fragmented glycocalyx, or (5) progressive multiple frosted shadows in both lungs is defined as a progressive SERMIE, which is considered a high-risk condition for progression to DIC or ARDS with poor prognosis (Figure 11).

## 10. Severe Inflammation-Induced Vascular Endothelial Damage

Post-mortem examination of the tissues of 3 patients who died of COVID-19: a post-renal transplant patient with coronary artery disease and hypertension, an obese patient with diabetes and hypertension, and a hypertensive patient, revealed traces of viral entry into vascular endothelial cells (presence of viral bodies), aggregation of inflammatory cells, and the presence of endothelial cells [6]. In addition, apoptosis and pyotosis (inflammation-induced programmed cell death) have been observed [6]. This “COVID-19 endotheliopathy” induces systemic vascular endothelial dysfunction, especially microcirculatory dysfunction, which has been implicated in the development of various complications.

The VEGLX is responsible for maintaining vascular homeostasis [74], including regulation of vascular permeability and microvascular tonus, prevention of microvascular thrombosis, and regulation of leukocyte adhesion [71]. In case of sepsis, the VEGLX may be involved in the regulation of inflammation-related enzymes such as metalloproteinase, heparanase, and hyaluronidase. Considering the fact that microvascular endothelial damage is difficult to assess using routine imaging, delays in diagnosis and treatment due to missed early microvascular damage are likely to make it even more difficult to predict the severity of the disease in COVID-19 patients.

Systemic damage to the vascular glycocalyx, which makes up a delicate layer, causes increased transport of proteins and water out of the vessels, that is, extravascular leakage of plasma components. In sepsis, the VEGLX is impaired and its layer becomes thinner, triggering excessive permeability of microvessels and causing interstitial edema in various organs [23,56]. Systemic shedding of the VEGLX can be led by serious infections, sepsis, hemorrhagic shock, burns, traumatic brain injury [57]. It occurs rapidly in fatal medical conditions such as traumatic endotheliosis, which is a mortality-related syndrome [55]. Patients with a variety of underlying diseases have chronic systemic VEGLX disorders due to a complex mechanism, but once these patients are infected with SARS-CoV-2, COVID-19-induced SIRME occurs in the process of the development of a variety of serious complications such as ARDS [21], DIC, Kawasaki disease shock syndrome, microvascular thrombosis, and arrhythmias (Figure 11).

## 11. Kawasaki Disease Shock Syndrome and VEGLX

Kawasaki disease is an acute febrile systemic vasculitis occurring primarily in children younger than 5 years of age, where systemic vasculitis is especially observed in small and medium-sized arteries. Since Kawasaki disease exhibits seasonal, temporal, and regional patterns, infectious agents are thought to be the causative or precipitating factor in Kawasaki disease [75]. Serological tests have reported that HCoV-229E, a type of coronavirus, is involved in the development of Kawasaki disease [76]. The exact mechanism of Kawasaki disease development is unknown, and for coronary artery aneurysms, it is believed to be a complex interaction of genetic factors, infection, and immunity [77]. For coronary artery aneurysms, it has been reported in males, intravenous immunoglobulin (IVIG) non-responders with high neutrophil/lymphocyte ratios, cases of inotropic drug use, and cases of heart failure. Significantly more coronary artery aneurysms occur in cases with abdominal pain and neurological symptoms [78].

An increase in Kawasaki disease or Kawasaki disease-like illness has been extensively reported in countries around the world occurring at the same time as the COVID-19 outbreaks. A severe subtype of Kawasaki disease, Kawasaki disease shock syndrome, is a rare complication of Kawasaki disease and is associated with a risk of serious sequelae and death [79]. Previous reports have shown that of 187 consecutive people diagnosed with Kawasaki disease, 13 (7%) met the definition of Kawasaki disease shock syndrome. Kawasaki disease shock syndrome is characterized by more severe proinflammatory cytokine production and tends to be prone to IVIG nonresponsiveness and coronary artery abnormalities [80]. Among severe COVID-19, characteristics consistent with toxic shock syndrome with abdominal pain and gastrointestinal symptoms similar to those of Kawasaki disease shock syndrome have been identified, as an increase in the number of children with the disease has been warned.

It has been shown that in the acute phase of Kawasaki disease, circulating levels of VEGLX (syndecan-1 and hyaluronic acid) are significantly elevated, and serum hyaluronic acid is the most useful prognostic biomarker for predicting future onset or exacerbation of coronary artery lesions in Kawasaki disease [46]. Serum levels of soluble syndecan-1, one of the major core proteins expressed in the VEGLX, are thought to reflect vascular endothelial damage and inflammation in Kawasaki disease [81]. Considering the common pathogenesis of Kawasaki disease and COVID-19, VEGLX damage has been suggested to be a biomarker in Kawasaki disease, and glycocalyx-associated biomarkers will likely contribute to the early detection of severe cases of COVID-19 in children and young adults, whose incidence of the disease is rare, but are prone to severe illnesses once they develop. Research on the development of biomarkers and new therapeutic strategies for predicting the onset of severe COVID-19 and COVID-19 patients who present with Kawasaki disease shock syndrome-like symptoms is expected to be promoted.

## 12. Characteristics of COVID-19

**Figure 12:**
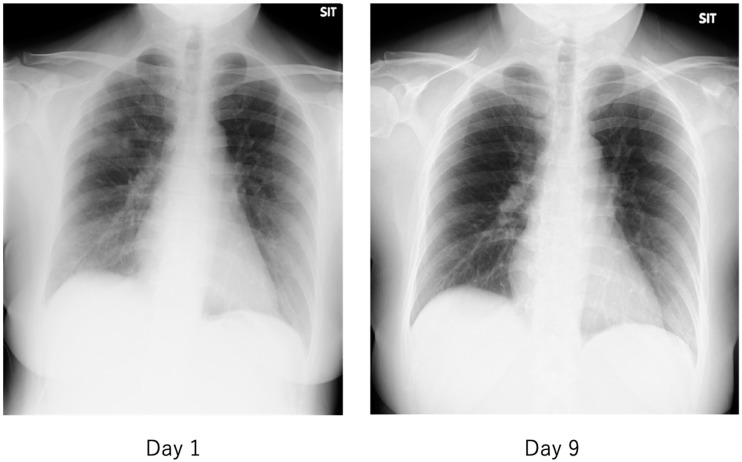
Chest radiograph of a patient with mild COVID-19. The fever of 38 °C lasted only half a day, and the next day, cough and nasal discharge were noted. A polymerase chain reaction (PCR) test for SARS-CoV-2 in a nasopharyngeal swab was performed 2 days after the fever was positive, and the patient was admitted to our hospital 2 days later. On admission, the patient had a low-grade fever, cough, nasal discharge, and conjunctival hyperemia, and chest radiographs showed multiple faint frosted shadows in the bilateral lung fields (**left**). With follow-up observation alone, the patient’s common cold-like symptoms were mild, and the abnormal shadows on the lungs had nearly disappeared by the ninth day of the disease (**right**). The patient was then discharged after two PCR tests confirmed negative results.

**Figure 13:**
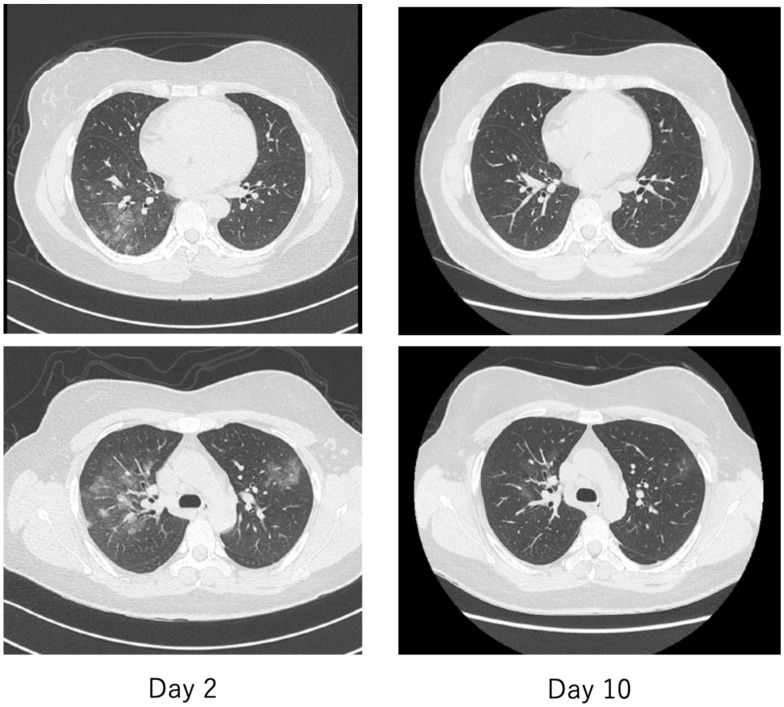
Chest computed tomography (CT) of a patient with mild COVID-19, the same patient as in Figure 12. On the second day, there were multiple faint frosted shadows bilaterally (**left**), but by the tenth day, the abnormal shadows had almost disappeared (**right**).

The features of COVID-19 that have not been seen in previous viral infections include asymptomatic pneumonia (Figure 12 and Figure 13), ARDS [82], and DIC [83] and their rapid progression, and sudden death associated with thromboembolism [84]. In particular, thromboembolism-related complications have been reported as an important complication closely related to the severity of COVID-19 patients, with deep vein thrombosis found in as many as 58% of autopsies of deceased COVID-19 patients [85]. Patients with COVID-19 are characterized by elevated blood D-dimer and fibrinogen degradation products (FDP) levels, prolonged prothrombin time, and the development of DIC, which are all associated with severe COVID-19. It was also a poor prognostic factor for patients [4]. In addition, a number of microvascular thromboses causing characteristic frostbite-like swelling of the hands and toes have been reported in patients with COVID-19 [86].

The criteria for severity of disease in COVID-19 are generally similar to those used in previous diseases, with patients requiring oxygen and either (1) admission to an intensive care unit (ICU), (2) requiring ventilatory management, or (3) leading to death. The hallmark imaging findings of COVID-19 are asymptomatic pneumonia detected by simple chest radiography or computed tomography (CT). As shown in Figure 12 and Figure 13, the frosty shadows are multiple and bilateral, and multiple rounds. Pale frosty shadows have been observed even in patients with mild COVID-19 who have only cold symptoms (e.g., mild fever, runny nose, and cough). These characteristic abnormal chest shadows in COVID-19 patients are not necessarily an indicator of the severity of the disease, and therefore, rapidly progressing signs of deterioration can be difficult to capture, especially in poorly symptomatic cases. It is difficult to foresee severe illness or sudden death in COVID-19.

## 13. Literature Search Results for VEGLX in COVID-19

The keywords “glycocalyx COVID-19”, “syndecan coronavirus”, hyaluronic acid COVID-19”, “heparanase COVID-19”, and “PBR COVID-19” were searched in databases such as PubMed, the WHO Global Health research database on COVID-19, and Japan Medical Abstracts Society. The research time frame for the database was until November 2020. The language was limited to English and Japanese. The titles and abstracts of all potentially relevant articles were read to determine their relevance. Full articles were also scrutinized if the titles and abstracts were unclear. Reference lists of identified articles were screened to ensure the completeness of the search.

**Figure 14:**
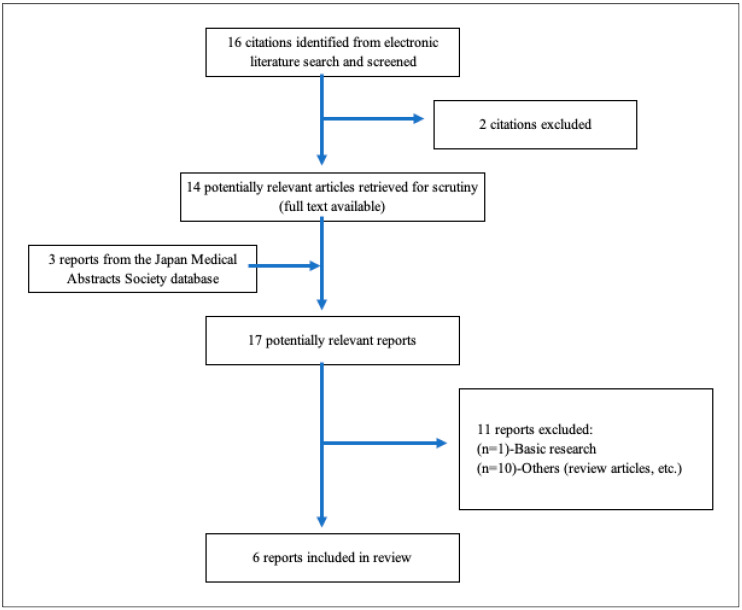
A flowchart depicting the selection process. A total of 16 studies were imported for title and abstract screening after removing duplicates, and 2 studies were retained. After a total of 11 studies were deemed eligible for full-text review, 6 reports [3,87,88,89,90,91] were included in Table 3.

As shown in Table 2, a total of 16 studies were included for the title and abstract screening, and 2 studies were excluded due to duplicates. After a total of 11 studies were not deemed eligible for full-text review, 6 reports [3,87,88,89,90,91] were included in the review. Figure 14 presents a flowchart depicting the selection process. Table 3 summarizes the characteristics of the included studies. All studies showed that circulating levels of glycocalyx or its kinases were increased in patients with severe COVID-19. Among them, only one study also revealed that VEGLX damage detected by PBR measurement was observed in severe patients with COVID-19. At present, only a few clinical studies have been conducted on COVID-19 patients; however, its effectiveness as a biomarker for predicting the severity of the disease and as an indicator of therapeutic efficacy in patients with COVID-19 could be very promising.

## 14. Future Outlook

The COVID-19 pandemic has fundamentally changed the lives of people around the world. Many cities have been sealed off, forcing people to stay in their homes, avoid contact with others, and scale back their economic activities. All non-essential activities are encouraged to stop, and even educational and work opportunities are being deprived by this epidemic. As far as we can surmise from this situation, it is getting to the point where we will never be able to return to the earlier situation again.

What we can do is to deeply understand the various aspects of the pathogenesis of viral infections, which will continue to evolve in the future, and to gather the wisdom of many to create effective countermeasures and the best way to survive together in the future. The VEGLX is a classic physical barrier common to many organisms, but this area of clinical research has not been well studied to date. It is precisely in the current challenging international climate that ideas from new areas of research must be shared to enrich our existing knowledge base.

## Figures and Tables

**Table 1:** SIRME and progressive SIRME.

Definition	Item	Specific Features (Meet Any)
SIRME (Meet the 3 items on the right at the same time)	Strong inflammation	Fever, high CRP, high inflammatory cytokines
	Vascular endothelial damage	Strong thrombotic tendency: high levels of D-dimer and FDP, and frostbite-like microvascular embolism. Increased vascular permeability: tissue edema
	Organ failure	Increased respiratory rate/hypoxia, high levels of lactate dehydrogenase and transaminase, and elevated cardiac deviation enzymes
Progressive SIRME (In addition to the above, meet any of the rights)		VEGLX damage: high blood levels of the fragmented glycocalyx, high PBR
		Progressive multiple frosted shadows in both lungs

SIRME, systemic inflammatory reactive microvascular endotheliopathy; CRP, C-reactive protein; FDP, fibrin/fibrinogen degradation products; VEGLX, vascular endothelial glycocalyx; PBR, perfused boundary regions.

**Table 2:** VEGLX damage in COVID-19.

	Coronavirus	SARS	COVID-19
glycocalyx	9 [87,88,89] (0/5/1)	4 (0/3/1)	12 [3,87,88,89] (0/7/1)
syndecan	4 [88] (0/1/2)	1 (0/1/0)	4 [88,90] (0/1/1)
hyaluronic acid	7 [88,91] (0/2/3)	4 (0/2/2)	8 [88,91] (0/3/3)
heparanase	3 [87] (0/1/1)	0	3 [87] (0/1/1)
PBR	0	0	1 [3] (0/0/0)

Numbers indicate the total number of articles [reference number] (study protocol article/review/non-clinical research). VEGLX, vascular endothelial glycocalyx; SARS, severe acute respiratory coronavirus; COVID-19, new coronavirus disease 2019; PBR, perfusion boundary region.

**Table 3:** VEGLX in patients with COVID-19.

	Patients (*n*)	Glycocalyx	Sheddase	Others
Buijsers B, et al. [87]	48		HPSE activity↑ HS↑	IL-6↑
Stahl K, et al. [89]	19	Syndecan-1↑	Hpa-1→ Hpa-2↓	Soluble Tie2↑ Angpt-1→ Angpt-2→
Fraser DD, et al. [88]	10	Syndecan-1↑ Hyaluronic acid↑		Soluble P-selectin↑
Hutchings SD, et al. [90]	30	Syndecan-1↑		IL-6↗
Ding M, et al. [91]	32	Hyaluronic acid↑		
Rovas A, et al. [3]	23	Syndecan-1↑ Hyaluronic acid↑ PBR↑		Angpt-1↑ Soluble Tie2↑ VEGF-A↑ Soluble Flt-1↑ TM↑ ACE2↑ VEGF-D↓ ADANTS13↓

(↑) shows significant upregulation, (↗) indicates mild upregulation, (↓) indicates downregulation, and (→) shows no significant changes. VEGLX, vascular endothelial glycocalyx; HPSE, heparinase; HS, heparan sulfate; IL-6, interleukin-6; Hpa, heparinase; Tie2, tunica interna endothelial cell kinase 2; Angpt, angiopoietin; VEGF, vascular endothelial growth factor; PBR, perfused boundary region; TM, thrombomodulin; ACE2, shed ectodomain of angiotensin-converting enzyme 2 receptor; ADAMTS13, a disintegrin and metalloprotease with a thrombospondin type 1 motif, member 13.
